# Is health-related quality of life associated with the risk of low-energy wrist fracture: a case-control study

**DOI:** 10.1186/1471-2474-10-80

**Published:** 2009-07-03

**Authors:** Gudrun Rohde, Anne M Mengshoel, Astrid K Wahl, Torbjorn Moum, Glenn Haugeberg

**Affiliations:** 1 Department of Rheumatology, Sorlandet Hospital, Kristiansand, Service box 416 4604 Kristiansand, Norway; 2 Institute of Nursing and Health Sciences, Medical Faculty, University of Oslo, Pb.1153 Blindern, 0316 Oslo, Norway; 3 Dept of Behavioural Sciences in Medicine, Medical Faculty, University of Oslo, Pb. 1111, Blindern, 0317 Oslo, Norway

## Abstract

**Background:**

Some risk factors for low-energy wrist fracture have been identified. However, self-reported measures such as health-related quality of life (HRQOL) have not been examined as potential risk factors for wrist fracture. The aims of this study were to compare HRQOL prior to a low-energy wrist fracture in elderly patients (≥ 50 years) with HRQOL in age- and sex-matched controls, and to explore the association between HRQOL and wrist fracture after adjusting for known risk factors for fracture such as age, weight, osteoporosis and falls.

**Methods:**

Patients with a low-energy wrist fracture (n = 181) and age- and sex-matched controls (n = 181) were studied. Shortly after fracture (median 10 days), patients assessed their HRQOL before fracture using the Short Form 36 (SF-36). Statistical tests included *t *tests and multivariate logistic regression analysis.

**Results:**

Several dimensions of HRQOL were significantly associated with wrist fracture. The direction of the associations with wrist fracture varied between the different sub-dimensions of the SF-36. After controlling for demographic and clinical variables, higher scores on *general health *(odds ratio (OR) = 1.31, 95% confidence interval (CI) = 1.10–1.56), *bodily pain *(OR = 1.18, 95% CI = 1.03–1.34) and *mental health *(OR = 1.39, 95% CI = 1.09–1.79) were related to an increased chance of being a wrist fracture patient rather than a control. In contrast, higher scores on *physical role limitation *(OR = 0.87, 95% CI = 0.79–0.95) and *social function *(OR = 0.65, 95% CI 0.53–0.80) decreased this chance. Significant associations with wrist fracture were also found for living alone (OR = 1.91, 95% CI 1.07–3.4), low body mass index (BMI) (OR = 0.92, 95% CI 0.86–0.98), osteoporosis (OR = 3.30, 95% CI 1.67–6.50) and previous falls (OR = 2.01, 95% CI 1.16–3.49).

**Conclusion:**

Wrist fracture patients perceive themselves to be as healthy as the controls before fracture. Our data indicate that patients with favourable and unfavourable HRQOL measures may be at increased risk of wrist fracture.

## Background

Low-energy fractures are common in the elderly population, and the wrist is one of the most common fracture sites [1-5]. By definition, low-energy fracture results from minimal trauma, e.g., falling from a standing position [6]. It is important to characterize and identify patients at high risk of low-energy fracture based on the presence of risk factors to understand better the potential complex mechanisms involved and thereby to develop preventive strategies to reduce fracture risk.

Low-energy fractures are related mainly to the event of falling. However, the reasons for falling may be explained by several factors. Obviously, a fall may relate to the activities people undertake and the environmental conditions in which activities are performed, as well as an individual's physical and mental capacities to meet the challenges of the activities. In ageing subjects health status may be impaired [1,4,5,7,8], and falling may relate to impaired physical health (e.g., reduced balance, inability to perform ordinary daily activities, or other diseases) or impaired mental health (e.g., cognition, inattentiveness, depression or anxiety). Moreover, because bone mineral density (BMD) declines with age [1], bones may have less strength to tolerate stress caused by falls. The importance of BMD at the time of fracture in explaining wrist fractures among elderly people is well documented [1,2,9,10], whereas physical and mental health only have been used as outcome parameters to assess recovery after fracture [7,8].

Health-related quality of life (HRQOL) may be seen as a patient's evaluation of his or her health status. Thus, HRQOL is defined as the individual's experience of his or her general state of health, such as physical, social and mental functioning, and well-being [11]. Self-reported health is supposed to capture the full array of a person's illness and possibly even symptoms of as yet undiagnosed diseases in preclinical stages [12,13]. Traditionally, HRQOL is measured using a questionnaire comprising a broad variety of health aspects such as bodily pain, general health, physical and social functioning, role functioning, mental health and vitality [14]. To understand the context of low-energy fractures in a broader sense, HRQOL evaluation may give a more complex perspective on the issue of possible fracture prevention. Strong risk factors for low-energy wrist fracture have been difficult to identify and are not widely studied, as wrist fractures often occur in relatively healthy elderly persons [1]. To our knowledge, no studies have examined self-reported health status as a potential risk factor before fracture, together with objective risk factors (e.g., BMD) in patients with a low-energy wrist fracture. Several studies reporting pre-injury HRQOL among patients with other types of low-energy fractures (e.g., hip fractures) do not include assessments of BMD and therefore lack an important health parameter [15-17].

This study examined pre-fracture HRQOL in low-energy wrist fracture patients by including self-reported physical and mental health and BMD. We first examined whether HRQOL shortly before fracture in elderly patients with low-energy wrist fracture differed from that of age- and sex-matched controls recruited randomly from the general population. We also explored whether HRQOL was independently associated with wrist fracture after adjusting for known risk factors for fracture such as age, weight, osteoporosis and falls.

## Methods

### Study design and patient recruitment

We used a case-control study design. Patients aged 50 years and older with low-energy wrist fractures were included. Sex- and age-matched control subjects were selected randomly from the general population in the catchment area of the wrist fracture patients.

Low-energy wrist fracture patients were recruited from a regional hospital in southern Norway in 2004 and 2005. The hospital is the only referral centre for orthopaedic trauma in the region. During the two-year period, 324 wrist fracture patients were treated at the hospital, and 249 of these patients were examined clinically at the osteoporosis centre. Among the 75 patients not examined at the osteoporosis centre, 14 patients were ineligible for BMD assessment because of poor mental or physical health, 13 patients were tourists, three patients were not invited for assessment for other reasons, and 45 patients declined to be assessed.

Among the 249 wrist fracture patients assessed at the osteoporosis centre, 181 patients were capable and willing to be enrolled in this study. Before patients were included in the study, we confirmed that the fracture met the definition of low-energy fracture and was not a result of high-energy trauma [6]. We excluded patients with confusion or dementia (as assessed by a nurse or doctor), serious infection, tourists and patients not capable of giving informed consent. Of the 68 patients assessed at the osteoporosis centre but not included in the study, 17 were not able to self-report their health status because of dementia or confusion. Another two patients were tourists who did not reside in the geographic area, three patients were not invited to participate in the study for other reasons and 46 patients declined to participate.

The median elapsed time between fracture and examination at the osteoporosis centre for participants in the study centre was 10 days (interquartile range 13). At this visit, BMD was measured and demographic, clinical and HRQOL data preceding fracture were assessed.

Age- and sex-matched controls were identified randomly in the national registry for the catchment area and invited by mail to participate in the study. The controls were identified consecutively. If a potential control refused to participate or did not respond to the invitation, a new control was invited. The mean (SD) time between the patient's inclusion and inclusion of the matched control was 4.9 (3.4) months. Overall, 131 potential controls refused to participate or did not respond to the invitation. We aimed for an age match of ± 1 year with the wrist fracture patients; however, this was a challenge in the cases aged 80 years and older. In these patients, we accepted a match of ± 5 years, except for one woman aged 96 years who was matched with a control aged 86 years. At the visit at the osteoporosis centre BMD was measured, and demographic, clinical and HRQOL data for the time preceding the visit was assessed.

### Demographic and clinical variables

At the osteoporosis centre, nurses recorded self-reported data for patients and controls on demographic details, height and weight (to compute body mass index (BMI)), regular exercise for at least 30 minutes three times a week or more (no/yes), and presence of co-morbidity (heart diseases, pulmonary diseases, neurological disorders, urogenital disorders, gastrointestinal disorders, endocrine disorders, inflammatory joint and connective tissue disorders, cancer and mental disorders). For co-morbidity, we also computed a total score for the number of the before mentioned diseases or conditions for each patient and control. Height and weight were measured for participants who did not know them. The osteoporosis nurses also recorded data about medication, smoking habits and the number of falls in the year before fracture or inclusion (controls).

For patients excluded from osteoporosis assessment, those who were unable or who declined to participate in the study, the only information available was age, sex and the reason for exclusion.

### Bone density measurements

BMD was assessed at lumbar spine L2-L4 and both hips using standardized measurement procedures and the same dual energy X-ray absorptiometry (DXA) equipment (General Electric, Lunar Prodigy). The machine was calibrated every day and was stable over the entire measurement period. The *in vivo *coefficient of variance for the measurement procedure performed by four trained nurses was 1.19% for lumbar spine L2-L4, 0.95% for the right total hip and 0.89% for the left total hip. The BMD measurements were expressed as T scores based on the reference value in the DXA machine provided by the manufacturer. Osteoporosis was defined as a T score ≤ -2.5 standard deviations (SD) at the hip and/or spine according to the World Health Organization definition of osteoporosis [6].

### Health-related quality of life assessment

The participants were asked to evaluate their HRQOL for the four weeks before the fracture (patients) or for the four weeks before inclusion (controls) using the Short Form 36 (SF-36) (The Medical Outcome Study). The SF-36 is a generic self-report questionnaire used to assess HRQOL and comprises 36 questions about various aspects of health. The questionnaire includes eight multi-item scales that reflect different health domains such as general health, bodily pain, physical function, physical role limitation, mental health, vitality, social function and emotional role limitation. One additional item assesses health transition over the previous year. The SF-36 scales were scored according to published scoring procedures, and each was expressed using values from 0 to 100, with 100 representing excellent health [14]. This questionnaire has shown satisfactory reliability and validity, and has been tested thoroughly for assessing psychometric properties in several countries, including Norway [14,18-20].

### Statistical analyses

Statistical analyses were performed using SPSS for Windows (version 16.0). For two-group comparisons, we used chi-squared tests for categorical variables and independent *t *tests for continuous variables. To examine the possible effects of less-than-perfect age matching between patients and controls (slightly younger oldest controls), age-adjusted unconditional logistic regression analysis was performed between the patient-control dichotomy and each of the demographic and clinical variables. To examine differences in HRQOL between wrist fracture patients and controls, we applied logistic regression analysis adjusting for age and sex [21]. The SF-36 domains were divided by a factor of 10 to estimate the odds ratio (OR). Differences between the groups of 5–10 points were regarded as modest and 10–20 as moderate with regard to their clinical significance [22].

Logistic regression analysis using the two comparison groups (fracture group and control group) as the dependent variable was used to select significant predictors (demographic, clinical and HRQOL variables; listed in Tables 1 and 2) to be retained in the final multivariate analysis of risk factors for wrist fracture. Most of these factors have been shown to be associated with the risk of low-energy fractures and were thereby potential risk factors to be retained in the final model [1,23]. The same eligible factors were included and retained following both forward entry and backward elimination (*p *< 0.05 for inclusion and *p *< 0.10 for retention). In the final model, we used the "enter" method of logistic regression analysis, which includes all the remaining independent variables in the model as one block, regardless of the level of significance obtained for individual variables. We also evaluated possible interactions between all pairs of independent variables, one pair at a time. The level of significance was set at *p *< 0.05.

**Table 1:** Demographics and clinical characteristics of the wrist fracture patients and the control group

	**Wrist fracture**	**Controls**	p*
	n = 181	n = 181	
**Demographics**			
Age (years)	66.9 (9.9)	66.8 (9.1)	0.885
Females	161 (89)	161 (89)	1.000
BMI (kg/m^2^)	25.4 (4.3)	26.6 (4.3)	**0.009**
Menarche (years)	13.9 (1.5)	13.6 (1.4)	**0.046**
Menopause (years)	48.9 (4.4)	49.5 (4.1)	0.228
Education			**0.029**
< 10 years	62 (37)	73 (40)	
11–13 years	70 (42)	52 (29)	
> 13 years	36 (21)	55 (31)	
Co-habiting	95 (53)	118 (66)	**0.014**
Regular exercise**	134 (74)	132 (73)	0.812
Current smoker	29 (16)	23 (13)	0.380
**Clinical characteristics**			
Heart diseases	56 (31)	62 (34)	0.501
Pulmonary diseases	24 (13)	13 (7)	0.056
Neurological diseases	14 (8)	17 (9)	0.573
Endocrine disorders	14 (8)	20 (11)	0.280
Gastrointestinal disorders	12 (7)	22 (12)	0.072
Urogenital disorders	5 (3)	1 (0.5)	0.100
Inflammatory joint and connective tissue disorders	43 (24)	50 (28)	0.400
Cancer	18 (10)	19 (11)	0.862
Mental disorders	7 (4)	11 (6)	0.333
Mean total score co-morbidity (range 0–6)	1.1 (1.1)	1.1 (1.1)	0.259
Current glucocorticoid treatment	12 (7)	3 (2)	**0.044**
Current calcium and/or vitamin D treatment	40 (22)	43 (24)	0.708
Current ART	26 (14)	11 (6)	**0.009**
Osteoporosis***	60 (33)	31 (17)	**< 0.001**
≥1 fall in the previous year	75 (47)	54 (37)	0.089
Previous fracture(s)	97 (54)	82 (46)	0.153

Continuous variables are presented as mean (SD) and group variables as numbers and (%).

* Chi-squared analysis was used to compare categorical data, and an independent *t *test was used to compare continuous variables.

Bold *p *values indicate significant differences between the groups.

** Exercise more than 30 minutes three times a week.

*** Osteoporosis at the total hip or lumbar spine L2-L4 or both.

BMI, body mass index; ART, anti-resorptive treatment, a specific osteoporosis treatment comprising oestrogens, biphosphonates, or selective oestrogen-receptor modulators.

**Table 2:** Differences in HRQOL (SF-36) between controls and wrist fracture patients, adjusted for age and sex.

SF-36 domain*	**Controls** **n = 181**	**Wrist fracture** **n = 181**		
			***p***	**OR per 10 points**

Bodily pain	71.8 (2.1)	73.6 (2.1)	0.575	1.02 (0.95–1.10)
General health	73.0 (1.8)	76.1 (1.8)	0.180	1.07 (0.97–1.18)
Physical function	82.2 (1.7)	83.8 (1.7)	0.490	1.04 (0.94–1.14)
Physical role limitation	80.0 (3.1)	68.4 (3.0)	**0.006**	0.93 (0.88–0.98)
Mental health	83.9 (1.2)	85.8 (1.2)	0.216	1.09 (0.95–1.26)
Social function	91.4 (1.7)	84.0 (1.7)	**0.002**	0.85 (0.77–0.94)
Vitality	65.0 (1.7)	64.7 (1.6)	0.876	0.99 (0.91–1.09)
Emotional role limitation	85.9 (2.7)	78.5 (2.7)	**0.043**	0.94 (0.88–1.00)

Data are presented as mean (SE)

Bold *p *values indicate significant differences between the groups using logistic regression analysis after adjusting for age and sex.

* The score for each SF-36 domain ranges from 0 to 100, where 100 means perfect health. The SF-36 domain scores were divided by a factor of 10 to estimate the OR.

### Ethical and legal aspects

The study was approved by the Regional Committee for Medical Research Ethics and the National Data Inspectorate.

## Results

### Response

The 181 wrist fracture patients included in this study were significantly younger than the patients who were excluded (mean = 76.2, SD = 11.5, *p *< 0.001) and the patients who declined to participate (mean = 71.8, SD = 11.2, *p *< 0.001). The excluded patients included 44 women (85%), and 73 women (79%) declined to participate. The 181 controls included in this study did not differ significantly with regard to age from the 131 potential controls who declined to participate (mean = 67.7, SD = 9.7, *p *= 0.406). The potential controls who declined to participate comprised 110 women (84%).

### Demographic and clinical characteristics in wrist fracture patients and controls

Differences in demographic and clinical characteristics between the wrist fracture patients and controls are shown in Tables 1. The wrist fracture patients had significantly lower BMI than the controls (*p *= 0.009). More wrist fracture patients were living alone (*p *= 0.014) and currently using more glucocorticoids (*p *= 0.044) and more anti-resorptive osteoporosis treatments (e.g., biphosphonates) (*p *= 0.009). The female patients had later menarche than the female controls (*p *= 0.046). Osteoporosis at one or both of the total hip sites or lumbar spine L2-L4 was found in 33% of the wrist fracture patients and 17% of the controls (*p *< 0.001) (Table 1). These associations persisted after adjusting for age when using the wrist fracture patients/controls dichotomy as a dependent variable in a series of logistic regression analyses with all of the demographic variables, clinical variables and SF-36 scales.

### Health-related quality of life in fracture patients and controls

Age- and sex-adjusted differences in HRQOL between wrist fracture patients and controls are shown in Table 2. The wrist fracture patients had significantly lower mean (standard error) scores than the controls for physical role limitation (mean = 68(3) vs mean = 80(3), *p *= 0.006), social function (mean = 84(2) vs mean = 91(2), *p *= 0.002) and emotional role limitation (mean = 79(3) vs mean = 86(3), *p *= 0.043). However, the differences seem to be of modest clinical significance (Table 2).

### Risk factors for low-energy wrist fracture

With regard to HRQOL, low-energy wrist fracture was observed more frequently among participants with significantly higher controlled scores for the following domains, expressed as OR with 95% confidence interval (CI): general health (OR = 1.31; 95% CI = 1.10–1.56), bodily pain (OR = 1.18; 95% CI = 1.03–1.34) and mental health (OR = 1.39; 95% CI = 1.09–1.79), while participants with low scores for physical role limitation (OR = 0.87; 95% CI = 0.79–0.95) and social function (OR = 0.65; 95% CI = 0.53–0.80) also had an increased risk of fracture. These are all rather weak statistical effects (see additional file 1). Furthermore, the risk of low-energy wrist fracture was also significantly related to low BMI (OR = 0.92; 95% CI 0.86–0.98), living alone (OR = 1.91; 95% CI 1.07–3.4), osteoporosis (OR = 3.30; 95% CI = 1.67–6.50) and one or more falls in the previous year (OR = 2.01; 95% CI = 1.16–3.49) (Table 3).

**Table 3:** Risk factors of low-energy wrist fractures assessed by logistic regression analysis

	**OR (95% CI)**	***p***
**Demographics**		
Age (per year)	0.97 (0.94–1.00)	0.055
BMI (kg/m^2^)	0.92 (0.86–0.98)	**0.013**
Living alone (n/y)	1.91 (1.07–3.40)	**0.028**
**SF-36 HRQOL**		
General health (per 10 points)	1.31 (1.10–1.56)	**0.003**
Bodily pain (per 10 points)	1.18 (1.03–1.34)	**0.012**
Physical role limitation (per 10 points)	0.87 (0.79–0.95)	**0.004**
Mental health (per 10 points)	1.39 (1.09–1.79)	**0.009**
Social function (per 10 points)	0.65 (0.53–0.80)	**< 0.001**
**Clinical characteristics**		
Osteoporosis in hip and/or spine (n/y)	3.30 (1.67–6.50)	**0.001**
≥1 fall(s) the last year (n/y)	2.01 (1.16–3.49)	**0.013**

Adjusted odds ratios (OR) (95% confidence interval (CI)) and *p *values. The SF-36 domain scores were divided by a factor of 10 to estimate the OR.

BMI, body mass index; n/y, no or yes answer; HRQOL, health-related quality of life.

Interaction terms between pairs of independent variables, which were tested one pair at a time, revealed no significant interactions between the independent variables in the logistic regression analyses.

## Discussion

The wrist fracture patients reported HRQOL before fracture at a level similar to the controls. Several dimensions of HRQOL were independently associated with increased risk of wrist fracture. However, the direction of the associations with wrist fracture varied between the different sub-dimensions of HRQOL. Higher scores on the SF-36 domains of general health, bodily pain and mental health, and lower scores on physical role limitation and social functioning were associated with an increased risk of wrist fracture. Our data indicate that patients with favourable and unfavourable HRQOL measures may be at increased risk of wrist fracture.

Our finding that favourable health in some SF-36 domains is associated with increased risk of wrist fracture contradicts the current opinion that only individuals with impaired health are at increased risk of low-energy fracture. One explanation may be that elderly people in good health lead a more active lifestyle, which may increase the risk of falling [1,4,5]. However, our HRQOL results seem to show a duality because we also found that individuals with impaired physical function were at increased risk of fracture. Kelsey et al. [4] concluded that wrist fractures often occur as a result of a fall in women with low BMD who are relatively healthy and active and have good neuromuscular function.

Our study identified BMI, osteoporosis and falls as independent risk factors for wrist fracture and agrees with the results of other studies [1,2,4,10,24]. Furthermore, we found that living alone was independently associated with increased risk of wrist fracture. As in our study, a lower rate of cohabitation in fracture patients has been reported previously [25]. This suggests that behavioural and psychological factors associated with living alone, which are also reflected in the HRQOL, might influence the risk of falls and fractures [26-28].

Our data suggest that a complex mix of circumstances and factors can increase or decrease the risk of low-energy fracture in the elderly. The clinical implications of our findings are equivocal because the association between the SF-36 domains and wrist fractures were inconsistent and use of SF-36 is not feasible in routine daily care. We emphasize that our results should not be used to recommend restriction of physical activity in elderly persons because the overall beneficial health effects of physical activity and an active lifestyle in elderly individuals are indisputable [29].

The significance of some differences in SF-36 domains between the wrist fracture patients and controls was not striking. Thus, the observed differences may be attributed to random or selection biases for both fracture patients and controls. To validate our control group, we compared the controls with normative Norwegian national data for the SF-36 domains [30]. Across all the SF-36 domains, controls had higher age- and sex-adjusted scores compared with the Norwegian normative data (*p *< 0.05). One could argue that the national population-based SF-36 norms might have provided better control values for our wrist fracture patients. However, this was a case-control study with patients and controls gathered from the same geographical area, and we used data from the matched controls for comparisons. Regardless of whether we compare with national norms or with the case controls, our results run counter to the hypothesis that low HRQOL is associated with low-energy wrist fractures.

Our study has some limitations that should be considered when interpreting the findings. The collection of HRQOL data retrospectively, i.e., after fracture, is a possible limitation because ideally such data would be collected before the fracture occurred. However, it is almost impossible to collect HRQOL data prospectively for a population with a specific injury, and alternative methods rely on pre-injury recall, as used in this trauma study and other studies [15,17,31,32]. Retrospective evaluations can be biased by recall problems and response shifts due to fracture [21,33,34]. To minimize this problem, it is recommended that HRQOL assessments should be performed with the shortest possible time lag after the fracture event, which we aimed for in our study [21,23]. The elapsed time from fracture to assessment was relatively short and most patients completed the pre-fracture HRQOL questionnaire within the first two weeks after fracture. Thus, it seems unlikely that the patients would have forgotten about their HRQOL immediately before and at the time of the fracture [35]. Patients who have experienced a recent change in health are more likely to make accurate responses than are controls [21]. To minimize further the limitations of our study design, the questionnaire was administered with a clear instruction that the patient should think of the period before the fracture. For our study, a prospective study design would also have limitations because HRQOL may change between the time of data capture and fracture and thus may no longer be valid for the time of fracture.

The controls were asked to report their HRQOL as it was prior to their visit to the osteoporosis centre. We could argue that controls also should have had a time lag of reporting their HRQOL, e.g., relate the questions to the time preceiding two weeks ago. On the other hand, the elapsed time between fracture and examination at the osteoporosis centre in the patients varied, and it was difficult to achieve a perfect match to reach the same time lag [34]

Furthermore, both patients and controls were asked to report objective data based on the preceding period or year before fracture or inclusion (controls), and both groups might be influenced by recall bias [21,34]. On the other hand, objective data are less likely to be influenced by events such as a fracture, and objective data in both patients and controls therefore would be quite comparable.

Both patients and controls self-reported their co-morbidities and other diseases and conditions. The participants' hospital records might have been a more reliable source, but hospital records also have their shortcomings. Furthermore, self-report was considered the most ethically correct approach. BMD measurements were carried out after the fracture, which is unlikely to influence the BMD measurement. The sites of BMD measurements were hip and spine, and BMD would not change significantly over a period of two weeks.

Only 56% of the wrist fracture patients referred to the hospital were included in this study. Inclusion of only those patients willing or capable of self-reporting their health status may have underestimated the importance of HRQOL as a risk factor for wrist fracture and the difference in HRQOL between wrist fracture patients and controls. Furthermore, the controls included in our study also comprised elderly persons willing to participate in the study. A selection bias towards the healthiest elderly might arguably occur. However, our data give us limited information regarding the controls who were unwilling to participate, and the assumption that it was the healthiest among the eligible controls who chose to participate in the study cannot be verified. Despite these limitations, a major strength of our study is that the patients included were recruited from the only referral centre for orthopaedic trauma in the region. Thus, we were able to account for all patients who were referred and treated at the hospital.

The inconsistent results observed in our study might be caused by the design, the retrospective recall used to assess HRQOL and covariates, and differences in sampling fractions for cases and controls [21]. It is possible that our findings should be regarded as restricted to elderly persons within the geographical area of our cases and controls. On the other hand, it must be regarded as a strength that patients and controls have been recruited from the same geographical area. As previously discussed, both retrospective and prospective designs to assess HRQOL and covariates have their shortcomings, and achieving identical sampling fractions for both cases and controls is difficult. Therefore, the design and methods used in this study seem to be appropriate to answer our research questions.

## Conclusion

Our data support the contention that wrist fracture patients perceive themselves to be quite healthy compared with controls from the general population. The association between wrist fracture and HRQOL was inconsistent, and favourable scores for some SF-36 domains were associated with an increased risk of wrist fracture, whereas other SF-36 domains exhibited the opposite pattern. Although several dimensions of HRQOL were related to an elevated risk for low-energy wrist fracture, our data suggest that the utility of HRQOL as an indicator of risk of fracture is limited in clinical practice because of the inconsistent direction of the associations for different sub-dimensions. Our study underlines the role of biological mechanisms, including low BMI, osteoporosis and previous falls, in increasing the risk of wrist fracture. In addition, behavioural and psychological risk factors associated with living alone should be considered. Further studies of HRQOL in wrist fracture patients are warranted, especially studies that include larger sample sizes, older patients and patients with greater impairment of physical and mental health.

## Abbreviations

ART: anti-resorptive treatment, a specific osteoporosis treatment comprising oestrogens, biphosphonates, or selective oestrogen-receptor modulators; BMD: bone mineral density; BMI: body mass index; CI: confidence interval; DXA: dual-energy X-ray absorptiometry; HRQOL: health-related quality of life; OR: odds ratio; SF-36: Short Form 36; SD: standard deviation; SE: standard error; WHO: World Health Organization.

## Competing interests

The authors declare that they have no competing interests.

## Authors' contributions

GR was involved in all part of this study, including data collection and analysis, and took the main responsible for writing the manuscript. GR initiated this paper as a part of a larger study of fracture patients. AMM supervised GR during the analysis and drafting of the paper. AKW supervised GR during the analysis and drafting of the paper. TM provided statistical advice. GH was the principal investigator for the research program in patients with low-energy wrist and hip fracture, and supervised GR during the analysis and drafting of the paper. All authors critiqued revisions of the paper and approved the final manuscript.

## Pre-publication history

The pre-publication history for this paper can be accessed here:



## Supplementary Material

Additional file 1 **The significance of the OR units in the SF-36 domains**. The SF-36 domains have been divided by a factor of 10 to estimate the OR, and the significance of the OR has been explained by an example. Click here for file
