# Supplementary material for: Is health-related quality of life associated with the risk of low-energy wrist fracture: a case-control study
Source: BMC Musculoskelet Disord. 2009 Jul 3;10:80. doi: 10.1186/1471-2474-10-80 (PMC2714004; doi:10.1186/1471-2474-10-80)
Supplement: Additional file 1 — The significance of the OR units in the SF-36 domains. The SF-36 domains have been divided by a factor of 10 to estimate the OR, and the significance of the OR has been explained by an example. [file 1471-2474-10-80-S1.doc]

**Appendix:**

**The significance of the OR units in the SF-36 domains**

The size of the OR (above or below unity) of course indicates the strength of the association, but the numerical value of the OR depends on the skewness of the dependent variable and the metric used for a continuous independent variable. To illustrate: with a dependent variable showing a 50/50 distribution (as in the present case) and a continuous variable with units corresponding to one-tenth of a 0–100 scale, an odds ratio of 1.35 (corresponding to an odds ratio of about 0.75 for an inverse association) would indicate an increase (decrease) in the proportion of cases of about 7% for every unit (i.e., 10-point) increase (decrease) in the SF-36 subscale. Similarly, an OR of 1.2 (corresponding to an OR of 0.85 for an inverse association) would indicate an increase (decrease) of about 4% of cases for each unit (10-point) increment (decrement) in the independent variable.
